# High Fat Diet and Polycystic Ovary Syndrome (PCOS) in Adolescence: An Overview of Nutritional Strategies

**DOI:** 10.3390/nu16070938

**Published:** 2024-03-24

**Authors:** Valeria Calcaterra, Vittoria Carlotta Magenes, Giulia Massini, Luisa De Sanctis, Valentina Fabiano, Gianvincenzo Zuccotti

**Affiliations:** 1 Department of Internal Medicine, University of Pavia, 27100 Pavia, Italy; 2 Pediatric Department, “Vittore Buzzi” Children’s Hospital, 20154 Milan, Italy; vittoria.magenes@unimi.it (V.C.M.); valentina.fabiano@unimi.it (V.F.); gianvincenzo.zuccotti@unimi.it (G.Z.); 3 Pediatric Endocrinology, Regina Margherita Children Hospital, 10131 Torino, Italy; giulia.massini@unimi.it (G.M.); luisa.desanctis@unito.it (L.D.S.); 4 Department of Public Health and Pediatric Sciences, University of Torino, 10131 Torino, Italy; 5 Department of Biomedical and Clinical Science, Università degli Studi di Milano, 20157 Milan, Italy

**Keywords:** polycystic ovary syndrome, high-fat diet, ketogenic diet, adolescents, nutritional strategies

## Abstract

Polycystic ovary syndrome (PCOS) is a multifaceted and heterogeneous disorder, linked with notable reproductive, metabolic, and psychological outcomes. During adolescence, key components of PCOS treatment involve weight loss achieved through lifestyle and dietary interventions, subsequently pursued by pharmacological or surgical therapies. Nutritional interventions represent the first-line therapeutic approach in adolescents affected by PCOS, but different kinds of dietary protocols exist, so it is necessary to clarify the effectiveness and benefits of the most well-known nutritional approaches. We provided a comprehensive review of the current literature concerning PCOS definition, pathophysiology, and treatment options, highlighting nutritional strategies, particularly those related to high-fat diets. The high-fat nutritional protocols proposed in the literature, such as the ketogenic diet (KD), appear to provide benefits to patients with PCOS in terms of weight loss and control of metabolic parameters. Among the different types of KD studies, very low-calorie ketogenic diets (VLCKD), can be considered an effective dietary intervention for the short-term treatment of patients with PCOS. It rapidly leads to weight loss alongside improvements in body composition and metabolic profile. Even though extremely advantageous, long-term adherence to the KD is a limiting factor. Indeed, this dietary regimen could become unsustainable due to the important restrictions required for ketosis development. Thus, a combination of high-fat diets with more nutrient-rich nutritional regimens, such as the Mediterranean diet, can amplify positive effects for individuals with PCOS.

## 1. Introduction

Diet plays a crucial role in the prevention and treatment of polycystic ovary syndrome (PCOS). Lifestyle modifications, adopting a healthy diet, and achieving or maintaining a healthy body weight are key therapeutic strategies for these patients. Dietary interventions for these patients should target specific objectives such as improving insulin resistance (IR) and metabolic and reproductive functions. This is the reason why lifestyle management, especially among adolescents, has consistently been recommended as the primary treatment method for PCOS [1,2].

The nutritional approach should be personalized to attain optimal outcomes for each patient. Currently, there is still no unique treatment for this condition; indeed, different kinds of dietary protocols exist, so it is necessary to clarify the effectiveness and benefits of the most well-known nutritional approaches. The high-fat nutritional protocols proposed in the literature appear to provide benefits to patients with PCOS. The term “high-fat diet” refers to consuming fat calories that make up 30% to 75% of the total daily caloric intake, up to reaching 90% in some dietary protocols. High-fat diets are typically defined as dietary patterns characterized by excessive consumption of saturated fatty acids and calories, characteristics that inevitably lead to weight gain and the onset of related conditions, including the exacerbation of PCOS symptoms [2,3]. It is the dietary pattern known as the “Western diet”, prevalent in industrialized countries and characterized by red and processed meats, sweetened meals and drinks, refined cereals, high-fat dairy products, and foods containing added sugars and fats [3,4]. The overconsumption of foods rich in calories but lacking in nutrients, combined with a low fiber content and excessive intake of added sugars and unhealthy fats, all negatively impact health [5].

Fortunately, not all high-fat diets contribute negatively to the development and progression of PCOS. Alternative dietary approaches have been assessed as therapeutic strategies, considering both the quality and quantity of fats. In particular, the literature highlights how the Mediterranean diet (MD) and the ketogenic diet (KD) have a positive impact on the treated condition.

The MD could stand out as one of the optimal nutritional strategies for PCOS treatment, as it provides antioxidants, substantial fiber, as well as vitamins, minerals, and other bioactive compounds. Another notable dietary advantage of the MD is the presence of healthy lipids, especially those obtained from olives, nuts, and fatty fish like salmon and sardines [6]. The added value of the MD lies in its high intake of omega-3 fatty acids, particularly eicosapentaenoic acid (EPA) and docosahexaenoic acid (DHA). The literature highlights how these fatty acids can help reduce the production of pro-inflammatory mediators, such as cytokines, chemokines, and eicosanoids, reducing inflammation. Additionally, EPA and DHA have an impact on the fluidity and integrity of cellular membranes, and they play an important role in oxidative stress [7]. The MD offers a range of elements that promote diverse and advantageous gut microbiota. Adhering to this dietary regimen can potentially enhance gut health, improve nutrient absorption, and reduce the incidence of various conditions, including type 2 diabetes (T2D), obesity, and cardiovascular diseases [8]. Indeed, the MD improves low-grade chronic inflammation, IR, and other metabolic unbalances typical of PCOS [9,10].

Another dietary regimen that has recently been studied in PCOS is the ketogenic diet (KD), aimed at controlling carbohydrate metabolism and IR [11,12]. This dietary approach is a high-fat dietary treatment characterized by adequate energy and protein intake and low carbohydrate content. In the KD, carbohydrates are restricted to less than 30–50 g/day [13]. This diet triggers ketosis and leads to the production of ketone bodies (acetone, acetoacetic acid, and β-hydroxybutyrate) by the liver, which compensate for the lack of sugars and become the primary energy source [12]. The KD has proven to be beneficial for several dysmetabolic conditions, such as T2D, cardiovascular disease, and PCOS [7]. Recently, studies on patients with PCOS revealed that the KD, through therapeutic ketosis, enhances various anthropometric and biochemical parameters, such as luteinizing hormone (LH), follicle-stimulating hormone (FSH), sex hormone-binding globulin (SHBG), insulin sensitivity, and HOMA index [13,14]. The KD was also shown to decrease androgenic production, and subsequently, the unregulated production of estrogens, contributing to an improvement in the LH/FSH ratio [13]. Several types of ketogenic diets (KD) have been investigated. Specifically, very low-calorie ketogenic diets (VLCKD), which restrict daily calorie intake to 700–800 kcal/day, can be regarded as an effective short-term intervention. They facilitate rapid weight loss while enhancing body composition and metabolic profile [13].

Long-term adherence to the KD has been indicated as a limiting factor because of the significant dietary restrictions required to induce ketosis, while the MD, which promotes heart health and possesses anti-inflammatory properties, could have a limited impact on weight loss [15]. Interestingly, a combination of the two nutritional approaches can amplify positive effects for individuals with reproductive system endocrine disorders [15].

PCOS is a complex and heterogeneous disorder that affects adolescents and women of reproductive age, and it is associated with significant reproductive, metabolic, and psychological consequences [16,17,18,19].

The pathogenesis of this disorder in adolescence is complex and results from genetic predisposition, epigenetic modification, and environmental factors [16,17,18]. The main endocrinological drivers of this condition are thought to be hyperandrogenism, hyperinsulinism, and IR [20], but other factors, such as the microbiome or epigenetics, also seem to be involved [21].

The diagnostic criteria for PCOS during adolescence pose another challenge in managing this disorder, as the criteria employed for diagnosis in adulthood may not always be applicable to adolescent girls [22] and precise criteria for adolescents have not been uniformly defined yet [23].

Early and personalized treatment is fundamental [24] for the possible PCOS lifelong metabolic [25] and reproductive implications [26] and important concerns [27].

The treatment options for PCOS vary depending on the specific manifestations being targeted, taking into account the patient’s phenotype and priorities. During adolescence, fundamental approaches to PCOS treatment include focusing on weight loss through lifestyle and dietary interventions, with pharmacological or surgical therapies as subsequent options if necessary [19].

We have provided a comprehensive overview of the existing literature concerning PCOS definition, pathophysiology, and therapeutic options, with a particular emphasis on nutritional strategies, especially high-fat diets.

## 2. Methods

**Figure 1:**
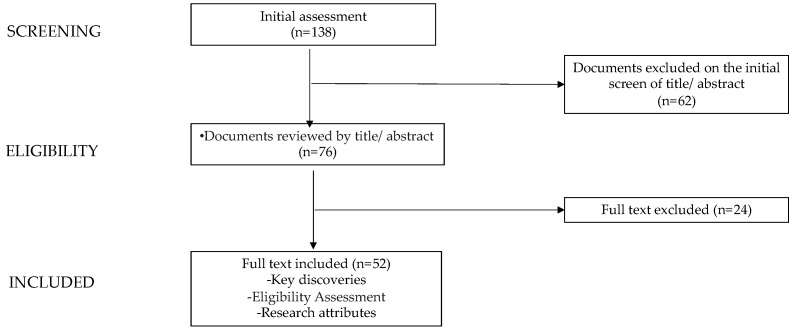
Process of manuscript selection and exclusion.

This narrative review aims to address nutritional strategies, with a focus on high-fat dietary regimens, in PCOS treatment in adolescents. The most pertinent original scientific papers, meta-analyses, clinical trials, and reviews published in the past decade in the English language were reviewed, given the increasing attention and research dedicated to this topic in recent years. PubMed, Scopus, and Web of Science were utilized for this research. The keywords used were the following: polycystic ovary syndrome in adolescents, insulin resistance in adolescent population, hyperandrogenism in adolescent population, obesity during adolescence, diet, nutrition, diet in polycystic ovary syndrome during adolescence, lifestyle intervention, ketogenic diet, Mediterranean diet, nutrition in PCOS during adolescence, very low-calorie ketogenic diets, low-calorie ketogenic diets, isocaloric ketogenic diets. The research was focused, when feasible, on adolescents (up to 18 years old) and young women. Starting from a total of papers (n = 138), the authors evaluated the abstract (n = 76) and subsequently scrutinized full-text documents to discern potentially relevant manuscripts (n = 52). Additionally, the reference lists of all papers were examined to identify relevant studies, which may have been included in the discussion even if they were published more than 10 years ago (Figure 1). The contributions were collected by VCM and GM and critically reviewed by VC, LdS, VF, and GZ. The draft resulting from this research was thoroughly discussed with all co-authors [28]. The final version was subsequently approved by all authors.

## 3. PCOS in Adolescents: Definition, Pathophysiology, and Therapeutic Strategies

### 3.1. Definition and Diagnosis

PCOS is a multifaceted and heterogeneous disorder that affects adolescents and women of reproductive age [16,17,18]. Reports on the epidemiology of PCOS in adolescents are scarce, and determining its prevalence accurately can be challenging due to the high occurrence of para-physiologic ovulatory dysfunction and the common ultrasound findings of micro-polycystic ovaries in adolescent girls [29]. Moreover, prevalence data are contingent upon the diagnostic criteria employed and the demographics of the observed population [29]. A recent systematic review and meta-analysis, which included nearly 150,000 adolescent girls worldwide, suggested that the prevalence of PCOS could be approximately 11% when evaluated using the Rotterdam criteria [19].

Three different sets of recommendations for the diagnosis of PCOS in adolescence have been published in recent years [24,30,31]. While these guidelines concur on the fundamental features of this condition, as outlined in Table 1, there are variations in the details among the three [32]. The primary diagnostic criteria include unexplained ovulatory dysfunction and evidence of hyperandrogenism (clinical and/or biochemical) [24,30,31].

The term ovulatory dysfunction indicates an abnormal menstrual pattern for one’s age. The specific menstrual abnormalities associated with ovulatory dysfunction are not consistently described, but the primary ovulatory dysfunctions considered across the three diagnostic guidelines are amenorrhea (either primary or secondary), oligomenorrhea, and excessive uterine bleeding [30]. Importantly, anovulatory cycles frequently occur in the initial years following menarche, making it common to experience menstrual irregularities in adolescent girls [24,30,31].

Indeed, the term primary amenorrhea refers to the absence of menarche by the age of 15 or within three years after the onset of breast development. Secondary amenorrhea, on the other hand, is defined as the absence of menstrual periods for more than 90 days after previously menstruating. Oligomenorrhea is characterized by an average cycle longer than 60 days during the first year post-menarche or longer than 45 days during the second and third years post-menarche. Excessive uterine bleeding is defined as bleeding that happens more frequently than every 21 days, lasts longer than 7 days, or is heavy [32].

Importantly, there is no unanimous consensus on the duration of persistence of menstrual irregularities after menarche, with recommendations varying between one year [24] and two years [30,31]. However, it is advised to monitor girls with menstrual irregularities during this period as they are considered ‘at risk for PCOS.’ Initiating treatment during this time may help reduce future comorbidities, irrespective of the definitive diagnosis [33].

Regarding hyperandrogenism, the primary clinical evidence of androgen excess is hirsutism, and according to the most recent criteria [24], even mild hirsutism is considered a sufficient criterion, whereas previous documents [30,31] suggested moderate to severe hirsutism. Severe inflammatory acne that does not respond to topical therapy is also considered an indication for testing androgen levels, according to the latest recommendations, although this clinical feature has not been included among the required diagnostic criteria [24]. Concerning biochemical assessment, all guidelines recommend measuring both total and free testosterone [30,33].

According to the three international expert conferences, neither polycystic ovary morphology nor obesity, IR, or severe cystic acne can be used as diagnostic criteria alone in adolescents [24,30,31,32]. Indeed, these clinical features are common during adolescence and can be evaluated in concert with the required features and the assessment of additional biomarkers, such as anti-mullerian hormone (AMH) or testosterone to dihydrotestosterone ratio (T/DHT), but should not be considered independently diagnostic [24,31].

Recently, Kiconco et al. performed a study to redefine reference values for the Rotterdam diagnostic criteria in adolescents (up to 8 years after menarche) [23]. The study group was composed of 226 post-menarchal Australian girls with a median age of 15 years. The authors gathered clinical, echographic, and biochemical data, establishing cut-off points for the free androgen index, free testosterone, menstrual length, and modified Ferriman–Gallwey score for clinical signs of hyperandrogenism [23]. Importantly, the cut-off values applied by the authors and the clinical hyperandrogenism score used for PCOS definition were lower than those commonly used in adults [23], highlighting the pertinent need to redefine PCOS diagnostic cut-offs in adolescents. The authors also underline that validation of these cut-offs is required in larger, multi-ethnic, and well-characterized adolescent cohorts [23].

### 3.2. Pathophysiology

Although PCOS is a frequent condition, its etiology, pathogenesis, and progression have not been fully clarified [34]. The pathogenetic mechanisms of this disorder in adolescence are intricate and arise from a combination of genetic predisposition, epigenetic modifications, and environmental factors [16,17,18].

In adolescents with PCOS, the process of follicular development is marked by elevated production of luteinizing hormone (LH), which in turn leads to increased androgen levels [34,35]. Furthermore, there is often insufficient rise in follicle-stimulating hormone (FSH), leading to decreased conversion of androgen to estradiol and arrest of follicular growth [36]. Furthermore, adolescents with PCOS commonly experience hyperinsulinemia and insulin resistance. Interestingly, these factors have been shown to correlate with hyperandrogenism, as elevated levels of androgens promote insulin resistance, and conversely, hyperinsulinemia promotes both androgen secretion in adipose tissue [37] and LH secretion from the pituitary gland [34].

Indeed, the major players in the pathogenesis of PCOS appear to be abnormal ovarian steroidogenesis and folliculogenesis [38,39], IR and hyperinsulinemia [38,40,41], and reproductive neuroendocrine dysfunction [41,42]. Although the precise pathophysiological roles of these factors have not yet been fully elucidated, recent research [41] has focused on assessing the pubertal ontogeny of PCOS, particularly examining developmental abnormalities in daughters of women with PCOS (PCOSd), girls with premature pubarche, and girls with obesity [17,41], all of whom are considered at risk for PCOS.

Individuals with PCOS have been observed to have higher levels of anti-Müllerian hormone (AMH), insulin, and androgen concentrations compared to controls [43,44,45]. Furthermore, these individuals exhibit higher ovarian volume [43] and increased estradiol and LH responses to GnRH stimulation [46], characteristics typical of patients with PCOS [24,30,31].

Girls with premature pubarche, one of the hallmarks of adrenarche, tend to have PCOS-like endocrinological dysfunction, such as hirsutism, oligomenorrhea, and biochemical hyperandrogenism upon GnRH stimulation [47]. Also, PP girls were found to have increased levels of AMH [48,49,50]. A possible link between PCOS and PP was hypothesized to be hyperinsulinism and IR, which work as triggers for androgen secretion by both adrenal glands and ovaries [51,52]. Indeed, IR was recently shown to have a prevalence of 70–80% in women with PCOS who are overweight and 30% in lean women [18,20].

**Figure 2:**
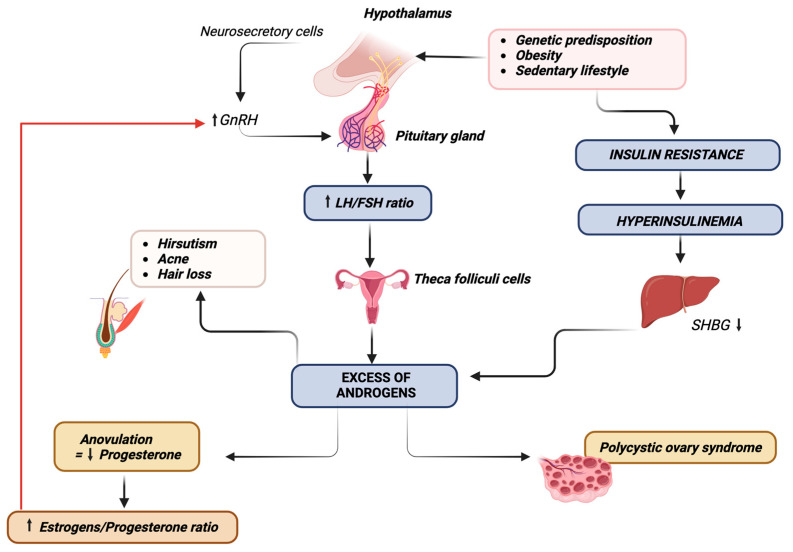
PCOS pathogenesis.

Based on studies conducted on girls at risk for PCOS, a hypothetical model for the pubertal development of PCOS suggests that initially, there may be a genetic predisposition to hyperinsulinemia. Subsequently, during adrenarche and puberty, this predisposition may result in an enhanced androgen steroidogenesis in response to stimulation by corticotropin and gonadotropins [20,41]. The hyperandrogenic environment, in turn, promotes post-pubertal neuroendocrine dysfunction, which impairs the gonadotropin-releasing hormone (GnRH) pulse generator. This impairment leads to increased luteinizing hormone (LH) release and decreased follicle-stimulating hormone (FSH) release [41]. These neuroendocrine unbalances support the progression to PCOS, further enhancing hyperandrogenemia and ovulatory dysfunction [41]. It is noteworthy to emphasize that IR in PCOS does not directly affect ovarian tissue. However, insulin can act as a co-gonadotropin through indirect mechanisms by enhancing luteinizing hormone (LH) action on theca cells. Additionally, insulin acts directly by inducing higher hypothalamic secretion of LH, resulting in increased production of dehydroepiandrosterone (DHEA) and androstenedione [18,20]. PCOS pathogenesis is depicted in Figure 2.

Importantly, studies have demonstrated that girls with obesity tend to have higher testosterone levels compared to non-obese individuals [45]. Obesity is observed in approximately 50% of patients diagnosed with PCOS [53], and research has shown that weight loss can lead to improvements in clinical features associated with PCOS [54]. Furthermore, childhood obesity has been linked to an increased risk of developing PCOS, both genetically and from a physiological standpoint [53,55], with IR being identified as a significant contributing factor [56].

Obesity and overweight were shown to also have a BMI-independent role in PCOS phenotype and metabolic disruption [53,57]. Indeed, in women with obesity, the disrupted folliculogenesis observed in PCOS arises from a combination of hyperandrogenism, hyperinsulinemia, and IR. Distinguishing between IR and hyperandrogenism in PCOS can be challenging, as these conditions often coexist and influence each other [57]. Interestingly, obesity is also a well-known trigger of low-grade inflammation, which enhances IR [53,57], and adipocytes secrete leptin that inhibits ovarian aromatase release, leading to a lower androgen-to-estrogen conversion rate, with an inhibitory effect on folliculogenesis [53,57].

Small for gestational age (SGA) babies, defined as neonates born with a weight <-2SDSs below the mean or <10th percentile for gestational age, represent another population considered at risk for the development of PCOS. They are predisposed to metabolic disturbances such as IR [58]. The mechanisms behind the association between SGA and PCOS remain poorly understood, and further studies should focus on a common origin between these conditions [58,59].

It is worth mentioning that gut microbiota, which is often altered in cases of obesity, appears to play a role in the pathogenesis of PCOS, suggesting a potential new therapeutic avenue [21]. Indeed, there has been significant research into the relationship between PCOS and changes in the microbiome in recent years, revealing notable differences in gut microbiota composition between patients with PCOS and control subjects [4,21,60,61,62,63]. Interestingly, besides alterations in the general composition of the microbiome, various studies have indicated an imbalance in specific bacterial species, such as Bacteroidetes and Firmicutes, in women with PCOS, which can impact the production of short-chain fatty acids and negatively affect metabolism [21,64].

Supplementation with prebiotics, probiotics, and synbiotics in women with PCOS seems to enhance various biochemical parameters. However, the precise mechanisms behind these effects remain unclear. Therefore, further research is required to elucidate the role of the microbiome and the potential therapeutic advantages of manipulating it through these agents in the treatment or prevention of PCOS [21].

Furthermore, the roles of genetics and epigenetics should be acknowledged. Although a specific inheritance pattern for PCOS has not yet been identified, there is evidence of familial aggregation among patients with PCOS [65,66], and polycystic ovarian morphology has been suggested to be inherited in an autosomal dominant manner [65]. Epigenetic mechanisms also appear to contribute to the development of PCOS, as animal models indicate that prenatal exposure to excess androgens may predispose individuals to PCOS [67]. Notably, Risal et al. recently demonstrated a five-fold increase in the risk of developing PCOS in mice born to mothers with PCOS [67].

### 3.3. Therapeutic Strategies

Since PCOS is associated with lifelong metabolic [25] and reproductive implications [26], as well as with other important clinical and psychological concerns [27], early and personalized treatment is fundamental [24]. In adolescents, no specific pharmacotherapy has been so far recommended by either the FDA or the EMA for the treatment of PCOS features, but different strategies are used to cope with PCOS symptomatology. The mainly utilized interventions have been divided into baseline and additional treatments [31].

Among the baseline interventions, the primary strategy is lifestyle modification, which includes adopting a healthy diet [68], engaging in sufficient physical activity, and, if needed, achieving weight loss [69]. These interventions have been linked to symptomatic improvements in girls with obesity or overweight [26], resulting in reduced androgen levels, improved menstrual regularity, and enhanced cardiometabolic health [31]. Additionally, within baseline interventions, local therapies and cosmetics are used to alleviate hirsutism and acne [34].

As for additional treatments, the main pharmacological options include estrogen-progestin (EP) contraceptive pills, metformin, and antiandrogens. Combined EP therapy is widely used in patients with PCOS due to its significant benefits in managing hirsutism, acne, and menstrual regulation [24,31]. However, recommendations suggest that further high-level studies are necessary before fully approving this therapy. Metformin, although not officially licensed for PCOS, is commonly used as an off-label insulin sensitizer. It has demonstrated benefits in girls with PCOS who are overweight as well as in normal-weight girls with PCOS and hyperinsulinemia. Metformin promotes weight loss, regulates menstrual cycles, improves acne, and aids in glycemic control, thus its widespread use despite lacking official approval for this syndrome [70]. Despite its clinical efficacy, metformin has not shown superiority when compared to lifestyle intervention alone or combined oral contraceptives in the treatment of PCOS [71].

Androgen receptor blockers (such as spironolactone, flutamide, and cyproterone acetate) and 5-alpha reductase inhibitors (like finasteride) are the two main options of anti-androgens commonly prescribed. There is no evidence suggesting preference for one type over the other, but spironolactone is the most commonly used in clinical practice [34]. Additionally, this drug has been shown to be more effective than metformin in regulating the menstrual cycle [72].

The guidelines recommend, when possible, a combination approach, based on the association of previously cited therapies, that can act synergistically [34].

Interestingly, the possible roles of vitamin D, omega-3 fatty acid, and probiotic supplementation in patients with PCOS have been evaluated [73]. The integration of vitamin D and probiotics was associated with diminished levels of testosterone and hirsutism, leading to psychological improvements in women affected by PCOS [74]. There are no definitive conclusions about the possible therapeutic role of these supplementations, and further studies in this field are needed, especially in adolescence [31].

To conclude, PCOS in adolescence remains an important concern, both from the diagnostic and the therapeutic points of view. Thus, additional studies are needed to improve diagnostic accuracy, avoid overdiagnosis or missed diagnosis, and improve treatment options.

## 4. High-Fat Diet and PCOS

The definition of a high-fat diet typically involves fat calories constituting 30% to 75% of the total daily caloric intake. However, some nutritional protocols may aim for even higher percentages, reaching up to 90%. Fat is the most energy-dense macronutrient in the diet, containing 9 kcal/g, as opposed to only 4 kcal/g for carbohydrates and proteins. Usually, high-fat diets are classified as dietary patterns characterized by excessive intake of saturated fatty acids and calories, which can lead to an increase in health problems, particularly heart diseases, weight gain, and obesity [75].

Cross-sectional studies suggest that higher fat intake is associated with impaired insulin sensitivity, although this association is primarily attributed to obesity [76,77]. Experiments involving overfeeding on fat indicate that an excess of fat intake reduces carbohydrate oxidation without any apparent alteration in fat oxidation. When carbohydrates are consumed in excess, fat deposition increases through de novo lipogenesis [76,77]. Consequently, high-fat diets can induce lipid accumulation in various tissues. Once the lipid storage capacity of non-adipose tissues reaches its limit, lipotoxicity may occur, leading to cellular dysfunction, cell death, and the development of obesity and its related diseases [76,77].

The enlargement of adipocytes, particularly in the abdominal region, has been shown to increase the release of pro-inflammatory cytokines from adipose tissue monocytes, in response to glucose and saturated fat intake [78,79]. This condition could worsen both the overall metabolic and reproductive outcomes in patients with PCOS. Central obesity plays a significant role in the onset of metabolic syndrome, with a reported prevalence of 43% in women with PCOS. This condition is marked by the presence of IR, hyperinsulinemia, and dyslipidemia [80,81]. Considering the association between PCOS, obesity, and IR, the primary recommendation for women with PCOS is weight management [13]. Studies conducted on patients with PCOS confirmed that weight loss can improve endocrine abnormalities such as hyperinsulinemia and reduction in androgen levels [76]. It is established that protracted consumption of a high-fat diet may result in the build-up of abdominal adipose tissue, consequently promoting the development of obesity, and it is recognized that obesity triggers IR and stimulates the production of testosterone from circulating androgens, simultaneously suppressing gonadotropin secretion. Under these circumstances, testosterone has been demonstrated to promote the accumulation of visceral fat in women with PCOS by inhibiting fat breakdown and facilitating fat synthesis [39,53].

The literature data have consistently shown a clear connection between dietary habits and the onset/progression of PCOS. Several reviews have indicated that individuals with this disorder tend to have high consumption of unhealthy foods, processed foods, saturated fats, carbohydrates, and animal proteins. Consequently, there has been emphasis on the importance of adopting dietary changes to improve the clinical condition of individuals with PCOS, which can lead to enhancements in hormonal health and metabolism [82,83].

In 2013, Altieri et al. observed that women with PCOS typically consumed higher amounts of high-glycemic index sweets, saturated fat, and cheese compared to healthy controls [84]. Conversely, beneficial foods for PCOS include low-fat dairy products, lean red meat, omega-3 fatty acid-rich fish, poultry, fish oils, olive oils, whole grains, and seeds such as pumpkin, sunflower, and almonds. Many of these foods are characteristic of special dietary patterns, such as the Mediterranean and ketogenic diets [85].

Various studies showed that not all high-fat diets negatively contribute to the onset and progression of PCOS. Alternative dietary approaches have been evaluated as therapeutic tools, taking into consideration both the quality and proportion of fats. Indeed, the nature of dietary fat influences insulin sensitivity and is associated with metabolic alterations. Epidemiological evidence and intervention studies have conclusively demonstrated that saturated fat exacerbates IR, whereas mono- and polyunsaturated fatty acids can enhance insulin sensitivity by modifying cell membrane composition [77]. Additionally, it is widely recognized that the effects of fats derived from animals differ. Omega-3 fatty acids, particularly eicosapentaenoic acid (EPA) and docosahexaenoic acid (DHA), have been associated with improved IR. Foods rich in omega-3 fatty acids typically include fatty fish. However, alpha-linolenic acid, which is the precursor of EPA and DHA, can be found in certain plant-based foods such as nuts and seeds. In women with PCOS, dietary supplementation with alpha-lipoic acid, N-acetylcysteine, and omega-3 fatty acids has been shown to possess antioxidant and anti-inflammatory properties, as well as to improve IR [86,87].

## 5. Nutritional Strategies for PCOS

The high-fat nutritional protocols proposed in the literature appear to provide benefits to patients with PCOS. In 2010, the MD was recognized as an intangible cultural heritage of humanity by UNESCO [6]. The macronutrient composition of this dietary pattern is 15% proteins, 30–35% lipids, and 50–55% carbohydrates, with less than 15% comprising simple sugars. Although the MD dietary model is characterized by fat accounting for up to 35% of total energy intake, the saturated fatty acids are less than 7–8%. The MD is regarded as the gold standard dietary model in preventive medicine because of its anti-inflammatory, antineoplastic, anti-obesogenic, and antioxidant properties [88]. It is a traditional dietary pattern of the Mediterranean region, characterized by a high abundance of plant-based foods such as cereals, legumes, nuts, fruits, vegetables, and herbs. It also involves a limited consumption of red and processed meat. This diet includes a moderate intake of fish, seafood, eggs, white meat, and dairy products, as well as a moderate consumption of alcohol (primarily wine during meals, where culturally acceptable). Olive oil is the primary source of added fat. The diet typically consists of five meals per day, including breakfast, lunch, dinner, and two snacks [6,89].

Given the close connection between PCOS and obesity, low-grade chronic inflammation, and IR, the Mediterranean diet (MD) stands out as one of the optimal non-drug strategies for PCOS treatment [9,10]. This dietary pattern offers antioxidants, significant fiber, as well as vitamins, minerals, and other bioactive compounds. Another notable advantage of the MD is the presence of healthy lipids, especially those obtained from olives, nuts, and fatty fish like salmon and sardines. These sources are rich in heart-healthy monounsaturated fats and are often used as substitutes for the saturated and trans fats found in fatty meats, cheeses, and packaged baked goods [6,89].

In 2022, Mei et al. [14] conducted a study to evaluate the therapeutic effect of a Mediterranean diet combined with a low-carbohydrate (LC) dietary model in 72 overweight patients with PCOS over a period of 12 weeks. Before and after the intervention, various parameters including body weight, body mass index (BMI), waist circumference, waist-hip ratio (WHR), body fat percentage, serum fasting insulin, fasting plasma glucose, HOMA index, quantitative insulin sensitivity check index (QUICKI), total cholesterol (TC), high-density lipoprotein cholesterol (HDL-C), low-density lipoprotein cholesterol (LDL-C), triglycerides (TG), total testosterone (TT), luteinizing hormone (LH), follicle-stimulating hormone (FSH), and prolactin (PRL) were evaluated.

This study was a randomized controlled clinical trial in which patients were randomly allocated to either the MD/LC diet or the low-fat (LF) diet, both involving energy restriction. The LF group adhered to a dietary plan where less than 30% of total caloric intake came from fat, with a daily fat intake of less than 40 g and a maximum of 10% saturated fat. In contrast, the MD/LC group participants had a maximum carbohydrate intake of less than 20%, with no more than 100 g of carbohydrates consumed throughout the day, along with increased intake of protein and fat. The average daily total calorie baseline was maintained constant for both dietary groups.

The findings of the study revealed that both the LF and MD/LC dietary models were successful in altering anthropometric parameters, reproductive endocrine levels, IR values, and lipid levels in patients with PCOS. However, the MD/LC dietary model demonstrated greater effectiveness, while the restoration of menstrual cycles was approximately similar in both groups [14].

The KD is a high-fat dietary treatment characterized by adequate energy and protein intake and low carbohydrate content. It is characterized by the restriction of carbohydrates, typically to less than 30–50 g/day [11]. The ketogenic diet is any dietary approach designed to trigger ketosis. The underlying mechanism of action of the ketogenic diet involves the production of ketone bodies (acetone, acetoacetic acid, and β-hydroxybutyrate) by the liver, which compensate for the lack of sugars, becoming the primary energy source [12].

The primary ketogenic condition is fasting; alternatively, this result can also be achieved with strongly hypocaloric diets or normocaloric, normoproteic diets that are strongly hypoglucidic and hyperlipidic. In these conditions, the body primarily relies on endogenous and exogenous lipids as an energy substrate [11]. During the initial three to four days, glucose is generated through glycogenolysis and gluconeogenesis. Subsequently, the combination of low glucose and insulin levels triggers the release of free fatty acids from adipose tissue. The liver synthesizes ketone bodies, serving as an alternative fuel source for extrahepatic organs such as muscles, the central nervous system, heart, kidneys, and others [11]. Various protocols for KD are documented in the literature. The specific calorie intake, macronutrient composition, and duration are tailored based on the intended goal to be achieved. In VLCKD, the daily caloric intake is typically limited to 700–800 kcal/day. There is no universal cut-off for either low-calorie ketogenic diet (LCKD) or isocaloric ketogenic diet (ICKD), as the determination of hypocaloric or isocaloric status is individualized based on total energy expenditure. In VLCKD, lipid intake can range from 30–40 g/day (often restricted to 10–20 g/day), and approximately 0.8 to 1.2 g/day of protein per kilogram of ideal body weight is typically supplied [90].

VLCKD is a multiphase diet, where the initial phase (up to 12 weeks) is ketogenic. Afterward, the patient undergoes a gradual transition, first to a low-calorie diet and then to a balanced diet on the Mediterranean model. Due to its very low caloric nature, it is advisable to supplement patients with micronutrients such as B-complex vitamins, vitamins C and E, minerals like potassium, sodium, magnesium, and calcium, as well as omega-3 fatty acids, in accordance with international recommendations [91,92].

The development of a ketogenic protocol involves calculating the KR (Ketogenic Ratio), i.e., the ratio of grams of fats to the sum of grams of proteins and carbohydrates (KR = g lipids/(g proteins + g sugars)). The initial phase of the ketogenic diet, i.e., the induction of ketosis, involves a dietary pattern with a low ketogenic ratio, typically 1:1 or 2:1, and may progress, if necessary, to a higher ketogenic ratio, such as 3:1 or 4:1. The latter is the ratio that induces the highest levels of ketosis, meaning composing the dietary plan with 4 g of fats for every gram of proteins + carbohydrates.

In clinical practice, different variations of the ketogenic diet are currently used:

- The classic ketogenic diet (CKD) is the most restrictive diet as it involves a fixed ketogenic ratio. Fats are primarily represented by long-chain triglycerides (LCT), constituting 90% of daily calories in the 4:1 ketogenic ratio diet, 87% in the 3:1 ketogenic ratio diet, and 82% in the 2:1 ketogenic ratio diet. Carbohydrate intake is highly limited, and protein is calculated to meet growth requirements. The CKD can be administered in three different ways, through the exclusive use of foods, in combination with functional foods formulated with a predetermined ketogenic ratio, or by the exclusive use of functional foods [93];
- The KD with medium-chain triglycerides (MCTKD) (70% lipids; 10% proteins; 20% carbohydrates), where 30–60% of the energy comes from oil based on medium-chain triglycerides (MCT), allows the production of more ketones per kilocalorie of energy compared to long-chain triglycerides. It enables a decrease in total fats and a higher intake of carbohydrates and proteins compared to the CKD;
- The Modified Atkins Diet (MAD) (64% lipids; 30% proteins; 6% carbohydrates), less restrictive compared to the two previous types, allows an intake of carbohydrates up to 20 g/day. It is based on a significant restriction of starchy foods, leading to a reduction in calorie intake and subsequent weight loss. The ketogenic ratio is around 1:1, and it is typically designed for the adolescent population to facilitate the management of the diet in daily life. It represents a valid alternative in case there is low compliance with the traditional method;
- The Low Glycemic Index Diet (LGIT) (60% lipids; 30% proteins; 10% carbohydrates) allows a carbohydrate intake of 40–60 g/day, restricted to foods with a low glycemic index (GI < 50). The aim of the LGIT is to prevent glycemic fluctuations and reduce insulin levels. It is the least restrictive among the four diets but also results in a lower average ketonemia.

Regardless of the type, ketogenic diets primarily include common foods that are rich in fats, both of plant and animal origin. Foods that are naturally high in fats and low in carbohydrates and have not undergone any industrial processing that alters their nutritional composition are referred to as “naturally ketogenic foods”: nuts, coconut, avocado, oils (in particular, extra virgin olive oil), seeds (pumpkin, flax, sunflower), olives, cocoa, and soy. Fats of animal origin are often accompanied by a component of saturated fats and cholesterol so, in recent years, there has been a promotion of using plant-based fats in ketogenic diets. However, animal fats imply the presence of animal proteins with high biological value, necessary for proper growth and, therefore, their presence is essential in the diet. Consequently, the inclusion of both categories of fats (animal and vegetable) remains advisable, as it promotes a greater variety of food choices and avoids the risk of nutritional deficiencies.

During the KD, it is necessary to drink more water than usual, at least in quantities equal to the daily water needs according to the reference intake levels to maintain adequate body hydration. The limited intake of fruits and vegetables in the ketogenic diet reduces the intake of water through food. On average, a balanced diet provides about 1000 mL of water through food, while the ketogenic diet has a reduced intake of approximately 250–300 mL of water per day, due to the specific choice of high-fat foods. The allowed beverages include tea and herbal teas without added sugar, coffee, and unsweetened drinks with sugar alcohols (xylitol, maltitol, sorbitol) or aspartame [91].

The KD is generally well-tolerated, but sometimes short-term side effects may occur, such as nausea, vomiting, diarrhea, loss of appetite, drowsiness, dehydration, and hypoglycemia. Long-term effects may include constipation, kidney stone formation, acidosis, hypocalcemia, hyperuricemia, dyslipidemia, osteopenia, and growth retardation in children.

On the other hand, certain conditions are absolute contraindications for the use of a ketogenic diet (KD). Pathophysiological conditions include comorbidities such as hepatic, renal, cardiac, and respiratory insufficiency; type 1 diabetes; recent myocardial infarction or cerebrovascular stroke; as well as severe psychiatric disorders. Furthermore, eating disorders, alcohol, and substance abuse are contraindications for a very-low carbohydrate ketogenic diet (VLCKD) [94].

KDs have been demonstrated to be effective for weight loss and to support the overall health of the female reproductive system thanks to their anti-inflammatory and antioxidant capacity [94].

Recent studies have shown that a VLCKD can lead to weight loss and improved insulin sensitivity in PCOS [95]. It is important to note that one of the advantages of being in a state of ketosis is the ability of ketone bodies to reduce appetite by inhibiting the release of cerebral neuropeptide Y and ghrelin [95]. Furthermore, the KD induces alterations in the metabolic framework and various molecular processes, offering several benefits [94]. These include heightened insulin sensitivity, decreased lipid synthesis, suppression of inflammatory pathways, protection against oxidative stress, and the initiation of autophagy and mitochondrion genesis [94,96]. It is noteworthy that mitochondria play an important role in oocyte quality, and their dysfunction has been observed with a consequent fertility compromise in woman with PCOS [94,96].

In 2020, Paoli et al. conducted a study aiming to assess the impact of a KD in women of childbearing age diagnosed with PCOS. They hypothesized that a modified KD, known as the KEMEPHY diet, a Mediterranean eucaloric ketogenic protocol of 1600/1700 kcal/day with the incorporation of some phytoextracts, would lead to improvements in body weight, plasma cholesterol, triglycerides, hyperinsulinemia, and hormonal outcomes. After twelve weeks of the dietary intervention, these patients experienced a significant decrease in body weight and BMI. Additionally, there was a reduction in fat mass and visceral adipose tissue, along with a significant improvement in IR. Furthermore, an enhancement in the hormonal profile (including LH, LH/FSH ratio, testosterone, SHBG, E2, and progesterone) was observed in these women [97].

In 2021, Cincione et al. also evaluated the beneficial effects of a ketogenic diet (with a protein intake of 1.1–1.2 g/kg/day of ideal body weight; maximum 30 g/day of carbohydrates; 30 g/day of lipids; 600 kcal/day) in 17 women with obesity and PCOS. The treatment plan, spanning a total of 45 days, followed KD criteria and employed a modified KD protocol referred to as “mixed ketogenic”. This “mixed ketogenic” diet included a daily protein intake partly from isolated whey protein powder derived from milk, which possesses a high biological value and a complete amino acid composition profile with minimal carbohydrate and fat content. Consistent with Paoli’s findings, the study revealed that the KD, leading to therapeutic ketosis, improved various anthropometric and biochemical parameters, including LH, FSH, SHBG, insulin sensitivity, and the HOMA index. It resulted in a reduction in androgenic production, and the simultaneous decrease in fat mass reduced the uncontrolled production of estrogens resulting from aromatization in adipose tissue due to excess androgens. This contributed to the improvement of the LH/FSH ratio [81].

Similar results were highlighted by Mavropoulos et al. in 2005, demonstrating a significant reduction in body weight, free testosterone levels, LH/FSH ratio, and fasting insulin after implementing a ketogenic diet regimen in affected patients [98].

In 2022, Magagnini et al. evaluated the effect of VLCKD on markers (SHBG, AMH, progesterone) suggested to be predictive of metabolic and ovulatory dysfunction in patients with PCOS and obesity (n = 25) for 12 weeks. After dietary intervention, patients showed a significant reduction in BMI, waist circumference, and HOMA index. Furthermore, AMH serum levels decreased, and progesterone and sex hormone-binding globulin (SHBG) increased significantly. The authors concluded that VLCKD could improve metabolic and ovulatory dysfunction in patients with PCOS [95]. A recent review conducted by Khalid et al. has confirmed the data just described, highlighting how a short-term ketogenic diet potentially improved hormonal imbalances commonly associated with PCOS [99].

Lifestyle interventions serve as the primary therapeutic approach in PCOS, and studies have demonstrated improvements in associated symptoms. There is no singular nutritional strategy for PCOS management; various nutritional approaches, such as the MD or the KD, can indeed yield similar improvements in nutritional status. Specifically, a VLCKD can be considered an effective dietary intervention for short-term treatment, promoting rapid weight loss and improvements in body composition, metabolic profile (including waist circumference, fat mass, blood glucose, HbA1c, and HOMA index), and insulin sensitivity—all fundamental aspects in the pathophysiology of PCOS [13].

Even though the benefits of the KD have been widely reported, long-term adherence to the KD is a limiting factor. Conversely, the MD, more nutrient-rich, promotes heart health and possesses anti-inflammatory properties, although its impact on weight loss may be limited. Thus, combining these two nutritional approaches can amplify positive effects for individuals with reproductive system endocrine disorders [15].

In Table 2 are summarized the literature relevant reports on high-fat nutritional protocols proposed to provide benefits to patients with PCOS.

Future research should explore the impact of varying types and proportions of fats on PCOS for the development of evidence-based and practical dietary intervention strategies for more effective prevention and management of PCOS.

## 6. Eating Disorders in Polycystic Ovary Syndrome

Recent evidence suggests that women with PCOS are at an increased risk of developing eating disorders (EDs), particularly binge eating disorder (BED) and bulimia nervosa (BN). Additionally, women experiencing BN and BED are more likely to exhibit polycystic ovaries [100,101,102]. BED is a condition characterized by rapid consumption of large amounts of food without purging and affects approximately 1–4% of adults and 1–2% of children and adolescents [103,104]. On the other hand, bulimia nervosa (BN) is characterized by rapid consumption of large amounts of food followed by purging and affects around 0.41–1.5% of adults [104].

The presence of EDs in individuals with PCOS can complicate treatment, particularly when it involves diet intervention and lifestyle modifications aimed at weight loss or weight management [31]. While weight loss in women with PCOS who are affected by obesity or overweight can lead to improvements in metabolic factors and menstrual function [31], a tailored nutritional plan focused on weight loss for an individual with an eating disorder may inadvertently reinforce harmful behaviors and worsen the ED [100,101]. Specifically, diet recommendations such as energy restriction may trigger or exacerbate symptoms of ED, including restrictive eating, purging, and excessive exercise [21,59]. Conversely, failure to achieve a desired weight may further exacerbate feelings of shame, guilt, and anxiety related to body image and food intake, worsening the symptoms of the eating disorder [105].

Interestingly, Lalonde-Bester et al. recently performed a review aiming to determine the prevalence of EDs and disordered eating in individuals with PCOS [100]. The authors confirmed that individuals with PCOS are at high risk of EDs. However, the degree of risk for different ED subtypes remains unclear [100]. The pathophysiology of EDs in PCOS involves metabolic, endocrine and psychological factors, such as body weight dissatisfaction, anxiety, and depression [106,107,108]. Moreover, it was hypothesized that women with PCOS may have altered eating and food-seeking behaviors due to concomitant hyperandrogenism [107]. Indeed a bidirectional relationship between metabolic dysfunctions, such as hyperandrogenism and hyperinsulinemia, and the development of EDs (BED and BN) in women with PCOS has been proposed [107]. Specifically, hyperinsulinemia and hyperandrogenism can predispose individuals to binging episodes, and binge eating behaviors may contribute to weight increase, hyperinsulinemia, and hyperandrogenism [107]. In addition, androgen excess, causing hirsutism and weight gain, may cause important body dissatisfaction, and dieting behaviors are often adopted to improve body image, cope with body dissatisfaction, and compensate for binge episodes [109]. The cycle of dieting-cravings promotes further binge episodes, which increase the risk of weight gain and dissatisfaction and may cause insulin unbalances [109].

It is crucial for clinicians to remain vigilant for EDs in women with PCOS, particularly in the context of weight management and lifestyle interventions [100,110]. If an ED is identified, patients should be promptly referred to an appropriate therapeutic pathway involving a clinical psychiatrist and/or psychologist and/or dietitian [100,107].

Moreover, although weight loss in women with obesity and PCOS can improve hormonal function, insulin sensitivity, and hyperandrogenism, it is essential to keep in mind the potential harm of placing emphasis on strict weight management in subjects suffering from an ED [100,105,111,112]. Further investigations on the links between PCOS and EDs and their mechanisms of reinforcement could be beneficial, leading to more effective preventive strategies and treatment modalities.

## 7. Limitations and Suggestions for Future Research

There are several limitations researchers encounter when seeking to understand the correlation between diet and PCOS. Some of these limitations include:

- Lack of long-term studies: the majority of studies exploring the relationship between diet and PCOS are short-term. Long-term studies are essential to evaluate the sustainability and enduring effects of dietary interventions on PCOS symptomatology;
- Lack of data in clinical trials conducted on adolescents: frequently studies are restricted to specific populations; in particular, few studies have been conducted on adolescent patients. The majority of studies are conducted on adult women;
- Lack of uniformity among the applied nutritional protocols;
- Few randomized case-control studies.

The methodology of this review has certain limitations, as the search for articles was confined to those published within the last 10 years and only articles in English were considered. Additionally, narrative reviews inherently possess limitations in terms of objectivity, completeness of the literature search, and interpretation of findings.

## 8. Conclusions

**Figure 3:**
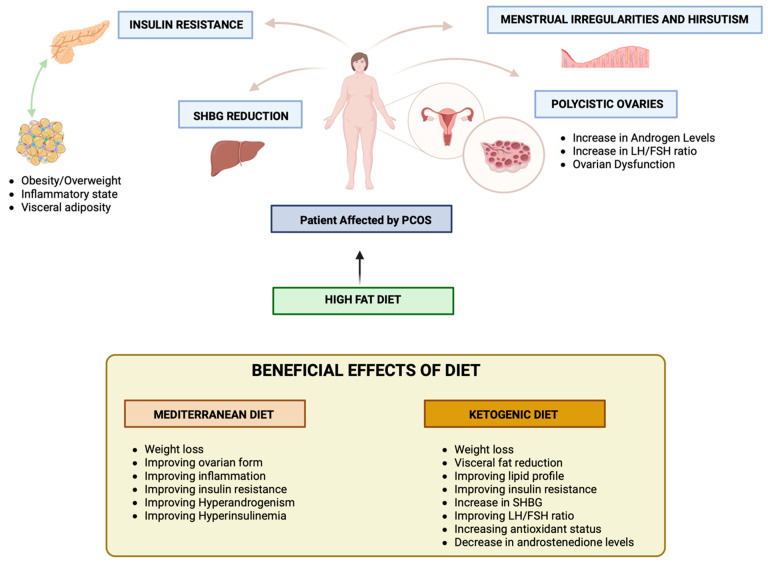
Effect of diet on Polycystic Ovary Syndrome.

Lifestyle interventions represent the first-line therapeutic approach in PCOS. There is no singular nutritional strategy for PCOS, but different approaches, such as the MD or KD, can result in improvements in nutritional status (Figure 3).

Specifically, the high-fat nutritional protocols proposed in the literature (such as KD) appear to provide benefits to patients with PCOS in terms of weight loss and control of metabolic parameters such as IR, glucose metabolism, lipid profile, and hormonal homeostasis [94,97,99]. Among the different types of KD studies, VCKD can be considered an effective dietary intervention for the short-term treatment of patients with PCOS, as it rapidly leads to weight loss, with improvements in body composition and metabolic profile [94,96].

Even though extremely advantageous, long-term adherence to the KD is a limiting factor. Indeed, this dietary regimen could become unsustainable due to the important restrictions required for ketosis development. Thus, a combination of high-fat diets with more nutrient-rich nutritional regimens (such as the MD) can amplify positive effects for individuals with reproductive system endocrine disorders, such as PCOS [15].

As evidence regarding the benefits of high-fat diets in adolescents remains limited, further studies are needed to gain a deeper understanding of their effects, especially in the long term. Future research should also aim to assess the impact of different types and ratios of fats on PCOS in order to develop evidence-based and practical dietary intervention strategies for more effective prevention and management of the condition.

## Figures and Tables

**Table 1:** Polycystic ovary syndrome (PCOS) diagnostic criteria in adolescents.

PCOS Diagnostic Criteria in Adolescents [24,31,32]
Required
Ovulatory dysfunction: abnormal menstrual pattern for the individual’s age or gynecologic age, which persists for 1–2 years. This pattern may include symptoms such as amenorrhea, oligomenorrhea, or excessive uterine bleeding. + Hyperandrogenism: biochemical (elevation of total/free serum testosterone) or clinical (moderate to severe hirsutism)
Not Recommended
Polycystic ovary morphology, Obesity, insulin resistance and/or hyperinsulinism, Severe acne, Biomarkers (T/DHT, AMH)

**Table 2:** Relevant high-fat nutritional protocols proposed in the literature to provide benefits for Polycystic Ovary Syndrome.

Reference	Study Type Duration	Population Age Range	Diagnostic Criteria Used for PCOS Diagnosis	Main Results	Main Limits
Altieri et al., 2013 [84]	Case-Control Study Dietary recall for 7 days and laboratory test examinations.	n = 100 women with PCOS and afeected by obesity or overweight. Age range: 18–45 y	Rotterdam criteria	High intake of high-glycemic index sweets, saturated fat, and cheese in patients with PCOS.	(1) The assessment of advanced glycosylated end products (AGE) content in foods was conducted only in small subsets of individuals with PCOS and controls; (2) The inclusion of individuals with overweight or obesity may have impacted the findings, given that the majority of studies indicating a correlation between AGE levels and PCOS have been carried out in women of normal weight
Mei et al., 2022 [14]	Randomized Controlled Clinical Trial 12-week duration.	n = 72 patients with overweight and PCOS. Age range: 16–45 y	Rotterdam criteria	The MED/LC diet restores menstrual cycles, improving anthropometric parameters, IR levels, and correcting endocrine disorders.	(1) Patient adherence to the dietary intervention was notably challenging, as PCOS patients encountered difficulty in adhering to a single dietary model for 12 consecutive weeks; (2) single-center trial; (3) all participants were Chinese patients; (4) the treatment period was limited to 12 weeks.
Paoli et al., 2020 [97]	Single-arm Controlled Clinical Trial 12-week duration.	n = 14 women with overweight and PCOS. Age range: age 19–35 y	Rotterdam criteria	After 12 weeks of KEMEPHY diet: a reduction of body weight, BMI, VAT, glucose, insulin blood levels, TG, TC, LDLc, LH/FSH ratio, LH total and free testosterone, and DHEAS. Estradiol, progesterone and SHBG increased.	(1) Inclusion of an oral glucose tolerance test for glucose and insulin would have provided additional insights into the metabolic effects of a ketogenic diet; (2) Small sample size and a single arm design.
Cincione et al., 2021 [81]	Controlled Clinical Trial Dietary treatment protocol for 45 days and final evaluation.	n = 17 women with obesity and diagnosis of PCOS. Age range: 18–45 y	Rotterdam criteria	KD improves LH, FSH, SHBG, insulin sensitivity and HOMA index, and reduces androgens.	(1) Small sample size; (2) Long term effects and adeherence to this dietary regiment could be problematic in PCOS patients.
Mavropoulos et al., 2005 [98]	Controlled Clinical Trial 6-month duration.	n = 11 with a BMI > 27 kg/m^2^ and with diagnosis of PCOS. Age range: 18–45 y	Not specified	LCKD led to significant improvement in weight, free testosterone %, LH/FSH ratio, and fasting insulin over a 24-week period.	(1) Weight loss can be a confounding factors when evaluating the effets of the dietary approach; (2) Hormonal measures were not taken at specified points during the menstrual cycle.
Magagnini et al., 2022 [95]	Controlled Clinical Trial 12-week duration.	n = 25 women with obesity and diagnosis of PCOS. Age range: 25–28 y	Rotterdam criteria	A reduction in BMI, WC, and HOMA index. A decrease in serum AMH levels. An increase of progesterone and SHBG levels.	(1) Small sample size and no control group; (2) Having no controls it is not possible to state that the benefits obtained are due to the KD alone or weight loss itself.
Khalid et al., 2023 [99]	Review Dietary interventions of at least 45 days in the studies included.	Single- or double-arm protocol. Age range of the studies included: 18–50 y	Rotterdam criteria	Short-term KD have shown potential in improving hormonal imbalances commonly associated with PCOS.	(1) Being this work a review and not a clinical trial it is not possible to drive clinical conclusions.

PCOS: Polycystic Ovary Syndrome; LF: Low-Fat Diet; MD: Mediterranean diet; LC: Low-Carbohydrate; IR: insulin resistance; KD: ketogenic diet; BMI: Body Mass Index; VAT: Visceral Adipose Tissue; TG: Triglycerides; TC: Total Cholesterol; LDL: Low-Density Lipoprotein Cholesterol; LCKD: low-carbohydrate ketogenic diet; SHBG: Sex hormone-binding globulin; VLCKD: Very low-carbohydrate ketogenic diet.

## References

[1] Faghfoori Z., Fazelian S., Shadnoush M., Goodarzi R. (2017). Nutritional Management in Women with Polycystic Ovary Syndrome: A Review Study. Diabetes Metab. Syndr..

[2] Che X., Chen Z., Liu M., Mo Z. (2021). Dietary Interventions: A Promising Treatment for Polycystic Ovary Syndrome. Ann. Nutr. Metab..

[3] Xiao Y.L., Gong Y., Qi Y.J., Shao Z.M., Jiang Y.Z. (2024). Effects of dietary intervention on human diseases: Molecular mechanisms and therapeutic potential. Signal Transduct. Target. Ther..

[4] Aziz T., Hussain N., Hameed Z., Lin L. (2024). Elucidating the Role of Diet in Maintaining Gut Health to Reduce the Risk of Obesity, Cardiovascular and Other Age-Related Inflammatory Diseases: Recent Challenges and Future Recommendations. Gut Microbes.

[5] Mente A., Dehghan M., Rangarajan S., McQueen M., Dagenais G., Wielgosz A., Lear S., Li W., Chen H., Yi S. (2017). Association of Dietary Nutrients with Blood Lipids and Blood Pressure in 18 Countries: A Cross-Sectional Analysis from the PURE Study. Lancet Diabetes Endocrinol..

[6] Serra-Majem L., Tomaino L., Dernini S., Berry E.M., Lairon D., Ngo de la Cruz J., Bach-Faig A., Donini L.M., Medina F.-X., Belahsen R. (2020). Updating the Mediterranean Diet Pyramid towards Sustainability: Focus on Environmental Concerns. Int. J. Environ. Res. Public Health.

[7] Aziz T., Khan A.A., Tzora A., Voidarou C., Skoufos I. (2023). Dietary Implications of the Bidirectional Relationship between the Gut Microflora and Inflammatory Diseases with Special Emphasis on Irritable Bowel Disease: Current and Future Perspective. Nutrients.

[8] Dinu M., Pagliai G., Casini A., Sofi F. (2018). Mediterranean Diet and Multiple Health Outcomes: An Umbrella Review of Meta-Analyses of Observational Studies and Randomised Trials. Eur. J. Clin. Nutr..

[9] Mirabelli M., Chiefari E., Arcidiacono B., Corigliano D.M., Brunetti F.S., Maggisano V., Russo D., Foti D.P., Brunetti A. (2020). Mediterranean Diet Nutrients to Turn the Tide against Insulin Resistance and Related Diseases. Nutrients.

[10] Barrea L., Arnone A., Annunziata G., Muscogiuri G., Laudisio D., Salzano C., Pugliese G., Colao A., Savastano S. (2019). Adherence to the Mediterranean Diet, Dietary Patterns and Body Composition in Women with Polycystic Ovary Syndrome (PCOS). Nutrients.

[11] Trimboli P., Castellana M., Bellido D., Casanueva F.F. (2020). Confusion in the Nomenclature of Ketogenic Diets Blurs Evidence. Rev. Endocr. Metab. Disord..

[12] Paoli A. (2014). Ketogenic Diet for Obesity: Friend or Foe?. Int. J. Environ. Res. Public Health.

[13] Barrea L., Verde L., Camajani E., Cernea S., Frias-Toral E., Lamabadusuriya D., Ceriani F., Savastano S., Colao A., Muscogiuri G. (2023). Correction: Ketogenic Diet as Medical Prescription in Women with Polycystic Ovary Syndrome (PCOS). Curr. Nutr. Rep..

[14] Mei S., Ding J., Wang K., Ni Z., Yu J. (2022). Mediterranean Diet Combined With a Low-Carbohydrate Dietary Pattern in the Treatment of Overweight Polycystic Ovary Syndrome Patients. Front. Nutr..

[15] Mentella M.C., Scaldaferri F., Ricci C., Gasbarrini A., Miggiano G.A.D. (2019). Cancer and Mediterranean Diet: A Review. Nutrients.

[16] Calcaterra V., Rossi V., Massini G., Casini F., Zuccotti G., Fabiano V. (2023). Probiotics and Polycystic Ovary Syndrome: A Perspective for Management in Adolescents with Obesity. Nutrients.

[17] Calcaterra V., Verduci E., Cena H., Magenes V.C., Todisco C.F., Tenuta E., Gregorio C., De Giuseppe R., Bosetti A., Di Profio E. (2021). Polycystic Ovary Syndrome in Insulin-Resistant Adolescents with Obesity: The Role of Nutrition Therapy and Food Supplements as a Strategy to Protect Fertility. Nutrients.

[18] Calcaterra V., Cena H., Sottotetti F., Hruby C., Madini N., Zelaschi N., Zuccotti G. (2023). Low-Calorie Ketogenic Diet: Potential Application in the Treatment of Polycystic Ovary Syndrome in Adolescents. Nutrients.

[19] Tay C.T., Hart R.J., Hickey M., Moran L.J., Earnest A., Doherty D.A., Teede H.J., Joham A.E. (2020). Updated Adolescent Diagnostic Criteria for Polycystic Ovary Syndrome: Impact on Prevalence and Longitudinal Body Mass Index Trajectories from Birth to Adulthood. BMC Med..

[20] Genazzani A.D., Genazzani R. (2023). Polycystic Ovary Syndrome as Metabolic Disease: New Insights on Insulin Resistance. Eur. Endocrinol..

[21] Giampaolino P., Foreste V., Di Filippo C., Gallo A., Mercorio A., Serafino P., Improda F.P., Verrazzo P., Zara G., Buonfantino C. (2021). Microbiome and PCOS: State-of-Art and Future Aspects. Int. J. Mol. Sci..

[22] Ortiz-Flores A.E., Luque-Ramírez M., Escobar-Morreale H.F. (2019). Polycystic Ovary Syndrome in Adult Women. Med. Clínica (Engl. Ed.).

[23] Kiconco S., Earnest A., Enticott J., Hart R., Mori T.A., Hickey M., Teede H.J., Joham A.E. (2023). Normative Cut-Offs for Polycystic Ovary Syndrome Diagnostic Features in Adolescents Using Cluster Analysis. Eur. J. Endocrinol..

[24] Teede H.J., Misso M.L., Costello M.F., Dokras A., Laven J., Moran L., Piltonen T., Norman R.J., Andersen M., Azziz R. (2018). Recommendations from the International Evidence-Based Guideline for the Assessment and Management of Polycystic Ovary Syndrome. Fertil. Steril..

[25] Ruan X., Li M., Mueck A.O. (2018). Why Does Polycystic Ovary Syndrome (PCOS) Need Long-Term Management?. Curr. Pharm. Des..

[26] Cooney L.G., Dokras A. (2018). Beyond Fertility: Polycystic Ovary Syndrome and Long-Term Health. Fertil. Steril..

[27] Ekramzadeh M., Hajivandi L., Noroozi M., Mostafavi F. (2020). Psychological Experiences of Adolescent Girls with Polycystic Ovary Syndrome: A Qualitative Study. Iran. J. Nurs. Midwifery Res..

[28] Bernardo W.M., Nobre M.R.C., Jatene F.B. (2004). Evidence-based clinical practice. Part II--Searching evidence databases. Rev. Assoc. Med. Bras. (1992).

[29] Bednarska S., Siejka A. (2017). The Pathogenesis and Treatment of Polycystic Ovary Syndrome: What’s New?. Adv. Clin. Exp. Med..

[30] Witchel S.F., Oberfield S., Rosenfield R.L., Codner E., Bonny A., Ibáñez L., Pena A., Horikawa R., Gomez-Lobo V., Joel D. (2015). The Diagnosis of Polycystic Ovary Syndrome during Adolescence. Horm. Res. Paediatr..

[31] Ibáñez L., Oberfield S.E., Witchel S., Auchus R.J., Chang R.J., Codner E., Dabadghao P., Darendeliler F., Elbarbary N.S., Gambineri A. (2017). An International Consortium Update: Pathophysiology, Diagnosis, and Treatment of Polycystic Ovarian Syndrome in Adolescence. Horm. Res. Paediatr..

[32] Rosenfield R.L. (2020). Perspectives on the International Recommendations for the Diagnosis and Treatment of Polycystic Ovary Syndrome in Adolescence. J. Pediatr. Adolesc. Gynecol..

[33] Kamboj M.K., Bonny A.E. (2017). Polycystic Ovary Syndrome in Adolescence: Diagnostic and Therapeutic Strategies. Transl. Pediatr..

[34] Trent M., Gordon C.M. (2020). Diagnosis and Management of Polycystic Ovary Syndrome in Adolescents. Pediatrics.

[35] Patel K., Coffler M.S., Dahan M.H., Malcom P.J., Deutsch R., Chang R.J. (2004). Relationship of GnRH-Stimulated LH Release to Episodic LH Secretion and Baseline Endocrine-Metabolic Measures in Women with Polycystic Ovary Syndrome. Clin. Endocrinol..

[36] Franks S., Stark J., Hardy K. (2008). Follicle Dynamics and Anovulation in Polycystic Ovary Syndrome. Hum. Reprod. Update.

[37] O’Reilly M., Gathercole L., Capper F., Arlt W., Tomlinson J. (2015). Effect of Insulin on AKR1C3 Expression in Female Adipose Tissue: In-Vivo and in-Vitro Study of Adipose Androgen Generation in Polycystic Ovary Syndrome. Lancet.

[38] Dumesic D.A., Oberfield S.E., Stener-Victorin E., Marshall J.C., Laven J.S., Legro R.S. (2015). Scientific Statement on the Diagnostic Criteria, Epidemiology, Pathophysiology, and Molecular Genetics of Polycystic Ovary Syndrome. Endocr. Rev..

[39] Rosenfield R.L., Ehrmann D.A. (2016). The Pathogenesis of Polycystic Ovary Syndrome (PCOS): The Hypothesis of PCOS as Functional Ovarian Hyperandrogenism Revisited. Endocr. Rev..

[40] Diamanti-Kandarakis E., Dunaif A. (2012). Insulin Resistance and the Polycystic Ovary Syndrome Revisited: An Update on Mechanisms and Implications. Endocr. Rev..

[41] Burt Solorzano C.M., McCartney C.R. (2021). Polycystic Ovary Syndrome: Ontogeny in Adolescence. Endocrinol. Metab. Clin. N. Am..

[42] McCartney C.R., Campbell R.E. (2020). Abnormal GnRH Pulsatility in Polycystic Ovary Syndrome: Recent Insights. Curr. Opin. Endocr. Metab. Res..

[43] Crisosto N., Echiburú B., Maliqueo M., Pérez V., Ladrón de Guevara A., Preisler J., Sánchez F., Sir-Petermann T. (2012). Improvement of Hyperandrogenism and Hyperinsulinemia during Pregnancy in Women with Polycystic Ovary Syndrome: Possible Effect in the Ovarian Follicular Mass of Their Daughters. Fertil. Steril..

[44] Crisosto N., Ladrón de Guevara A., Echiburú B., Maliqueo M., Cavada G., Codner E., Paez F., Sir-Petermann T. (2019). Higher Luteinizing Hormone Levels Associated with Antimüllerian Hormone in Postmenarchal Daughters of Women with Polycystic Ovary Syndrome. Fertil. Steril..

[45] Torchen L.C., Legro R.S., Dunaif A. (2019). Distinctive Reproductive Phenotypes in Peripubertal Girls at Risk for Polycystic Ovary Syndrome. J. Clin. Endocrinol. Metab..

[46] Crisosto N., Codner E., Maliqueo M., Echiburú B., Sánchez F., Cassorla F., Sir-Petermann T. (2007). Anti-Müllerian Hormone Levels in Peripubertal Daughters of Women with Polycystic Ovary Syndrome. J. Clin. Endocrinol. Metab..

[47] Ibáñez L., Potau N., Virdis R. (1993). Postpubertal Outcome in Girls Diagnosed of Premature Pubarche during Childhood: Increased Frequency of Functional Ovarian Hyperandrogenism. J. Clin. Endocrinol. Metab..

[48] Idkowiak J., Lavery G.G., Dhir V., Barrett T.G., Stewart P.M., Krone N., Arlt W. (2011). Premature Adrenarche: Novel Lessons from Early Onset Androgen Excess. Eur. J. Endocrinol..

[49] Paterson W.F., Ahmed S.F., Bath L., Donaldson M.D.C., Fleming R., Greene S.A., Hunter I., Kelnar C.J.H., Mayo A., Schulga J.S. (2010). Exaggerated Adrenarche in a Cohort of Scottish Children: Clinical Features and Biochemistry. Clin. Endocrinol..

[50] Broekmans F.J., Visser J.A., Laven J.S.E., Broer S.L., Themmen A.P.N., Fauser B.C. (2008). Anti-Müllerian Hormone and Ovarian Dysfunction. Trends Endocrinol. Metab..

[51] Rosenfield R.L. (1996). Evidence That Idiopathic Functional Adrenal Hyperandrogenism Is Caused by Dysregulation of Adrenal Steroidogenesis and That Hyperinsulinemia May Be Involved. J. Clin. Endocrinol. Metab..

[52] Ibáñez L., Díaz R., López-Bermejo A., Marcos M.V. (2009). Clinical Spectrum of Premature Pubarche: Links to Metabolic Syndrome and Ovarian Hyperandrogenism. Rev. Endocr. Metab. Disord..

[53] Glueck C.J., Goldenberg N. (2019). Characteristics of Obesity in Polycystic Ovary Syndrome: Etiology, Treatment, and Genetics. Metabolism.

[54] Anderson A.D., Solorzano C.M.B., McCartney C.R. (2014). Childhood Obesity and Its Impact on the Development of Adolescent PCOS. Semin. Reprod. Med..

[55] Day F., Karaderi T., Jones M.R., Meun C., He C., Drong A., Kraft P., Lin N., Huang H., Broer L. (2018). Large-Scale Genome-Wide Meta-Analysis of Polycystic Ovary Syndrome Suggests Shared Genetic Architecture for Different Diagnosis Criteria. PLoS Genet..

[56] Nokoff N., Thurston J., Hilkin A., Pyle L., Zeitler P.S., Nadeau K.J., Santoro N., Kelsey M.M. (2019). Sex Differences in Effects of Obesity on Reproductive Hormones and Glucose Metabolism in Early Puberty. J. Clin. Endocrinol. Metab..

[57] Zeng X., Xie Y., Liu Y., Long S., Mo Z. (2020). Polycystic Ovarian Syndrome: Correlation between Hyperandrogenism, Insulin Resistance and Obesity. Clin. Chim. Acta.

[58] Calcaterra V., Cena H., Regalbuto C., Vinci F., Porri D., Verduci E., Chiara M., Zuccotti G.V. (2021). The Role of Fetal, Infant, and Childhood Nutrition in the Timing of Sexual Maturation. Nutrients.

[59] Ibáñez L., Potau N., Francois I., de Zegher F. (1998). Precocious Pubarche, Hyperinsulinism, and Ovarian Hyperandrogenism in Girls: Relation to Reduced Fetal Growth. J. Clin. Endocrinol. Metab..

[60] Insenser M., Murri M., Del Campo R., Martínez-García M.Á., Fernández-Durán E., Escobar-Morreale H.F. (2018). Gut Microbiota and the Polycystic Ovary Syndrome: Influence of Sex, Sex Hormones, and Obesity. J. Clin. Endocrinol. Metab..

[61] Torres P.J., Siakowska M., Banaszewska B., Pawelczyk L., Duleba A.J., Kelley S.T., Thackray V.G. (2018). Gut Microbial Diversity in Women With Polycystic Ovary Syndrome Correlates With Hyperandrogenism. J. Clin. Endocrinol. Metab..

[62] Liu S., An Y., Cao B., Sun R., Ke J., Zhao D. (2020). The Composition of Gut Microbiota in Patients Bearing Hashimoto’s Thyroiditis with Euthyroidism and Hypothyroidism. Int. J. Endocrinol..

[63] Thursby E., Juge N. (2017). Introduction to the Human Gut Microbiota. Biochem. J..

[64] Thackray V.G. (2019). Sex, Microbes, and Polycystic Ovary Syndrome. Trends Endocrinol. Metab..

[65] Amin M., Gragnoli C. (2023). Genome-Wide Linkage and Association Study Identifies Novel Genes and Pathways Implicated in Polycystic Ovarian Syndrome. Eur. Rev. Med. Pharmacol. Sci..

[66] Vink J.M., Sadrzadeh S., Lambalk C.B., Boomsma D.I. (2006). Heritability of Polycystic Ovary Syndrome in a Dutch Twin-Family Study. J. Clin. Endocrinol. Metab..

[67] Risal S., Pei Y., Lu H., Manti M., Fornes R., Pui H.-P., Zhao Z., Massart J., Ohlsson C., Lindgren E. (2019). Prenatal Androgen Exposure and Transgenerational Susceptibility to Polycystic Ovary Syndrome. Nat. Med..

[68] Moran L.J., Ko H., Misso M., Marsh K., Noakes M., Talbot M., Frearson M., Thondan M., Stepto N., Teede H.J. (2013). Dietary Composition in the Treatment of Polycystic Ovary Syndrome: A Systematic Review to Inform Evidence-Based Guidelines. J. Acad. Nutr. Diet..

[69] Harrison C.L., Lombard C.B., Moran L.J., Teede H.J. (2011). Exercise Therapy in Polycystic Ovary Syndrome: A Systematic Review. Hum. Reprod. Update.

[70] Naderpoor N., Shorakae S., de Courten B., Misso M.L., Moran L.J., Teede H.J. (2015). Metformin and Lifestyle Modification in Polycystic Ovary Syndrome: Systematic Review and Meta-Analysis. Hum. Reprod. Update.

[71] Al Khalifah R.A., Florez I.D., Zoratti M.J., Dennis B., Thabane L., Bassilious E. (2021). Efficacy of Treatments for Polycystic Ovarian Syndrome Management in Adolescents. J. Endocr. Soc..

[72] Ganie M.A., Khurana M.L., Eunice M., Gulati M., Dwivedi S.N., Ammini A.C. (2004). Comparison of Efficacy of Spironolactone with Metformin in the Management of Polycystic Ovary Syndrome: An Open-Labeled Study. J. Clin. Endocrinol. Metab..

[73] Ostadmohammadi V., Jamilian M., Bahmani F., Asemi Z. (2019). Vitamin D and Probiotic Co-Supplementation Affects Mental Health, Hormonal, Inflammatory and Oxidative Stress Parameters in Women with Polycystic Ovary Syndrome. J. Ovarian Res..

[74] Sadeghi A., Djafarian K., Mohammadi H., Shab-Bidar S. (2017). Effect of Omega-3 Fatty Acids Supplementation on Insulin Resistance in Women with Polycystic Ovary Syndrome: Meta-Analysis of Randomized Controlled Trials. Diabetes Metab. Syndr. Clin. Res. Rev..

[75] Han Y., Wu H., Sun S., Zhao R., Deng Y., Zeng S., Chen J. (2023). Effect of High Fat Diet on Disease Development of Polycystic Ovary Syndrome and Lifestyle Intervention Strategies. Nutrients.

[76] Farshchi H., Rane A., Love A., Kennedy R.L. (2007). Diet and Nutrition in Polycystic Ovary Syndrome (PCOS): Pointers for Nutritional Management. J. Obstet. Gynaecol..

[77] Riccardi G., Rivellese A.A. (2000). Dietary Treatment of the Metabolic Syndrome--the Optimal Diet. Br. J. Nutr..

[78] Wu L.L.-Y., Dunning K.R., Yang X., Russell D.L., Lane M., Norman R.J., Robker R.L. (2010). High-Fat Diet Causes Lipotoxicity Responses in Cumulus-Oocyte Complexes and Decreased Fertilization Rates. Endocrinology.

[79] González F. (2015). Nutrient-Induced Inflammation in Polycystic Ovary Syndrome: Role in the Development of Metabolic Aberration and Ovarian Dysfunction. Semin. Reprod. Med..

[80] Norman R.J., Dewailly D., Legro R.S., Hickey T.E. (2007). Polycystic Ovary Syndrome. Lancet.

[81] Cincione R.I., Losavio F., Ciolli F., Valenzano A., Cibelli G., Messina G., Polito R. (2021). Effects of Mixed of a Ketogenic Diet in Overweight and Obese Women with Polycystic Ovary Syndrome. Int. J. Environ. Res. Public Health.

[82] Sedighi S., Amir Ali Akbari S., Afrakhteh M., Esteki T., Alavi Majd H., Mahmoodi Z. (2014). Comparison of Lifestyle in Women with Polycystic Ovary Syndrome and Healthy Women. Glob. J. Health Sci..

[83] Alomran S., Estrella E.D. (2023). Effect of Dietary Regimen on the Development of Polycystic Ovary Syndrome: A Narrative Review. Cureus.

[84] Altieri P., Cavazza C., Pasqui F., Morselli A.M., Gambineri A., Pasquali R. (2013). Dietary Habits and Their Relationship with Hormones and Metabolism in Overweight and Obese Women with Polycystic Ovary Syndrome. Clin. Endocrinol..

[85] Xenou M., Gourounti K. (2021). Dietary Patterns and Polycystic Ovary Syndrome: A Systematic Review. Maedica.

[86] Frias-Toral E., Garcia-Velasquez E., de Los Angeles Carignano M., Rodriguez-Veintimilla D., Alvarado-Aguilera I., Bautista-Litardo N. (2022). Polycystic Ovary Syndrome and Obesity: Clinical Aspects and Nutritional Management. Minerva Endocrinol..

[87] Günalan E., Yaba A., Yılmaz B. (2018). The Effect of Nutrient Supplementation in the Management of Polycystic Ovary Syndrome-Associated Metabolic Dysfunctions: A Critical Review. J. Turk. Ger. Gynecol. Assoc..

[88] Di Lorenzo M., Cacciapuoti N., Lonardo M.S., Nasti G., Gautiero C., Belfiore A., Guida B., Chiurazzi M. (2023). Pathophysiology and Nutritional Approaches in Polycystic Ovary Syndrome (PCOS): A Comprehensive Review. Curr. Nutr. Rep..

[89] Di Rosa C., Lattanzi G., Spiezia C., Imperia E., Piccirilli S., Beato I., Gaspa G., Micheli V., De Joannon F., Vallecorsa N. (2022). Mediterranean Diet versus Very Low-Calorie Ketogenic Diet: Effects of Reaching 5% Body Weight Loss on Body Composition in Subjects with Overweight and with Obesity-A Cohort Study. Int. J. Environ. Res. Public Health.

[90] Castellana M., Conte E., Cignarelli A., Perrini S., Giustina A., Giovanella L., Giorgino F., Trimboli P. (2020). Efficacy and Safety of Very Low Calorie Ketogenic Diet (VLCKD) in Patients with Overweight and Obesity: A Systematic Review and Meta-Analysis. Rev. Endocr. Metab. Disord..

[91] Muscogiuri G., Barrea L., Laudisio D., Pugliese G., Salzano C., Savastano S., Colao A. (2019). The Management of Very Low-Calorie Ketogenic Diet in Obesity Outpatient Clinic: A Practical Guide. J. Transl. Med..

[92] Moreno B., Crujeiras A.B., Bellido D., Sajoux I., Casanueva F.F. (2016). Obesity Treatment by Very Low-Calorie-Ketogenic Diet at Two Years: Reduction in Visceral Fat and on the Burden of Disease. Endocrine.

[93] Dowis K., Banga S. (2021). The Potential Health Benefits of the Ketogenic Diet: A Narrative Review. Nutrients.

[94] Camajani E., Feraco A., Verde L., Moriconi E., Marchetti M., Colao A., Caprio M., Muscogiuri G., Barrea L. (2023). Ketogenic Diet as a Possible Non-Pharmacological Therapy in Main Endocrine Diseases of the Female Reproductive System: A Practical Guide for Nutritionists. Curr. Obes. Rep..

[95] Magagnini M.C., Condorelli R.A., Cimino L., Cannarella R., Aversa A., Calogero A.E., La Vignera S. (2022). Does the Ketogenic Diet Improve the Quality of Ovarian Function in Obese Women?. Nutrients.

[96] Caprio M., Infante M., Moriconi E., Armani A., Fabbri A., Mantovani G., Mariani S., Lubrano C., Poggiogalle E., Migliaccio S. (2019). Very-Low-Calorie Ketogenic Diet (VLCKD) in the Management of Metabolic Diseases: Systematic Review and Consensus Statement from the Italian Society of Endocrinology (SIE). J. Endocrinol. Investig..

[97] Paoli A., Mancin L., Giacona M.C., Bianco A., Caprio M. (2020). Effects of a Ketogenic Diet in Overweight Women with Polycystic Ovary Syndrome. J. Transl. Med..

[98] Mavropoulos J.C., Yancy W.S., Hepburn J., Westman E.C. (2005). The Effects of a Low-Carbohydrate, Ketogenic Diet on the Polycystic Ovary Syndrome: A Pilot Study. Nutr. Metab..

[99] Khalid K., Apparow S., Mushaddik I.L., Anuar A., Rizvi S.A.A., Habib A. (2023). Effects of Ketogenic Diet on Reproductive Hormones in Women With Polycystic Ovary Syndrome. J. Endocr. Soc..

[100] Lalonde-Bester S., Malik M., Masoumi R., Ng K., Sidhu S., Ghosh M., Vine D. (2024). Prevalence and Etiology of Eating Disorders in Polycystic Ovary Syndrome: A Scoping Review. Adv. Nutr..

[101] Lee I., Cooney L.G., Saini S., Smith M.E., Sammel M.D., Allison K.C., Dokras A. (2017). Increased Risk of Disordered Eating in Polycystic Ovary Syndrome. Fertil. Steril..

[102] Lee I., Cooney L.G., Saini S., Sammel M.D., Allison K.C., Dokras A. (2019). Increased Odds of Disordered Eating in Polycystic Ovary Syndrome: A Systematic Review and Meta-Analysis. Eat. Weight Disord..

[103] Kjeldbjerg M.L., Clausen L. (2021). Prevalence of Binge-Eating Disorder among Children and Adolescents: A Systematic Review and Meta-Analysis. Eur. Child. Adolesc. Psychiatry.

[104] Galmiche M., Déchelotte P., Lambert G., Tavolacci M.P. (2019). Prevalence of Eating Disorders over the 2000–2018 Period: A Systematic Literature Review. Am. J. Clin. Nutr..

[105] Memon A.N., Gowda A.S., Rallabhandi B., Bidika E., Fayyaz H., Salib M., Cancarevic I. (2020). Have Our Attempts to Curb Obesity Done More Harm Than Good?. Cureus.

[106] Blay S.L., Aguiar J., Passos I.C. (2016). Polycystic Ovary Syndrome and Mental Disorders: A Systematic Review and Exploratory Meta-Analysis. NDT.

[107] Paganini C., Peterson G., Stavropoulos V., Krug I. (2018). The Overlap Between Binge Eating Behaviors and Polycystic Ovarian Syndrome: An Etiological Integrative Model. CPD.

[108] Burnatowska E., Wikarek A., Oboza P., Ogarek N., Glinianowicz M., Kocelak P., Olszanecka-Glinianowicz M. (2023). Emotional Eating and Binge Eating Disorders and Night Eating Syndrome in Polycystic Ovary Syndrome—A Vicious Circle of Disease: A Systematic Review. Nutrients.

[109] Goldschmidt A.B., Wall M., Loth K.A., Le Grange D., Neumark-Sztainer D. (2012). Which Dieters Are at Risk for the Onset of Binge Eating? A Prospective Study of Adolescents and Young Adults. J. Adolesc. Health.

[110] Dokras A., Stener-Victorin E., Yildiz B.O., Li R., Ottey S., Shah D., Epperson N., Teede H. (2018). Androgen Excess- Polycystic Ovary Syndrome Society: Position Statement on Depression, Anxiety, Quality of Life, and Eating Disorders in Polycystic Ovary Syndrome. Fertil. Steril..

[111] Ee C., Pirotta S., Mousa A., Moran L., Lim S. (2021). Providing Lifestyle Advice to Women with PCOS: An Overview of Practical Issues Affecting Success. BMC Endocr. Disord..

[112] Dilbaz B., Cınar M., Ozkaya E., Tonyalı N.V., Dilbaz S. (2012). Health Related Quality of Life among Different PCOS Phenotypes of Infertile Women. J. Turk. Ger. Gynecol. Assoc..

